# Low-Rank Matrix Recovery Approach for Clutter Rejection in Real-Time IR-UWB Radar-Based Moving Target Detection

**DOI:** 10.3390/s16091409

**Published:** 2016-09-01

**Authors:** Donatien Sabushimike, Seung You Na, Jin Young Kim, Ngoc Nam Bui, Kyung Sik Seo, Gil Gyeom Kim

**Affiliations:** 1 Department of Electronics and Computer Engineering, Chonnam National University, 77 Yongbong-ro, Buk-gu, Gwangju 61186, Korea; donatsabu6@gmail.com (D.S.); beyondi@jnu.ac.kr (J.Y.K.); 2 MOMED Solution, Gwangju 61008, Korea; buingocnam87@gmail.com (N.N.B.); momsolution@naver.com (K.S.S.); nayak3@naver.com (G.G.K.)

**Keywords:** UWB, moving target detection, background subtraction, matrix decomposition, low-rank, sparse, RPCA, augmented Lagrange multiplier, online processing, real-time processing

## Abstract

The detection of a moving target using an IR-UWB Radar involves the core task of separating the waves reflected by the static background and by the moving target. This paper investigates the capacity of the low-rank and sparse matrix decomposition approach to separate the background and the foreground in the trend of UWB Radar-based moving target detection. Robust PCA models are criticized for being batched-data-oriented, which makes them inconvenient in realistic environments where frames need to be processed as they are recorded in real time. In this paper, a novel method based on overlapping-windows processing is proposed to cope with online processing. The method consists of processing a small batch of frames which will be continually updated without changing its size as new frames are captured. We prove that RPCA (via its Inexact Augmented Lagrange Multiplier (IALM) model) can successfully separate the two subspaces, which enhances the accuracy of target detection. The overlapping-windows processing method converges on the optimal solution with its batch counterpart (i.e., processing batched data with RPCA), and both methods prove the robustness and efficiency of the RPCA over the classic PCA and the commonly used exponential averaging method.

## 1. Introduction

In recent years, Ultra-Wideband (UWB) radars have attracted a large amount of interest in research due to their wide variety of practical applications and their ability to operate in indoor environments and areas with poor visibility conditions. The most famous applications are the detection, localization and tracking of a moving target because of the interest for rescue, surveillance and security purposes. Some recent works proved that the UWB radar sensor networks can be used for improved accuracy while working within wireless environments with multipath, clutter, line-of-sight (LOS) blockage, excess propagation delays through materials, and other harsh conditions [1,2].

**Figure 1:**
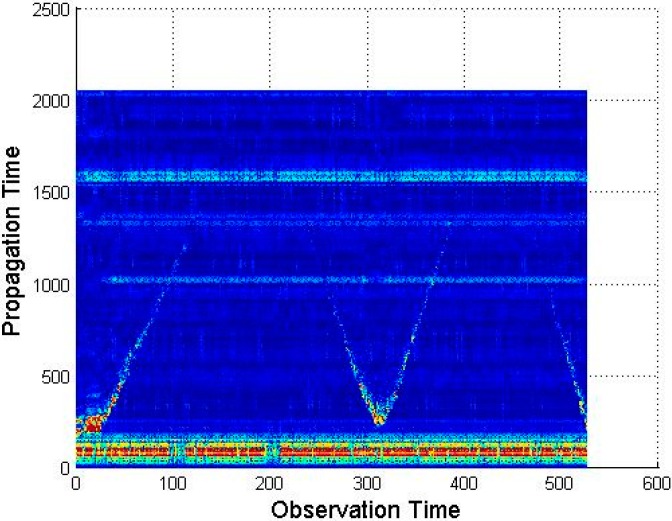
Raw data Radargram.

The basic idea behind UWB technology is to transmit impulses of electro-magnetic wave (EMW). It is not possible to create an ideal impulse in the real world, so UWB technology uses a pulse with a finite amplitude and very short duration, typically ∼0.5–1.5 ns, as a realistic approximation. This pulse will have a very wide frequency spectrum, hence the name Impulse-Radio UltraWideband (IR-UWB). After the radar transmits a pulse, it starts listening and the returned waveforms are recorded for a fixed interval of time. This interval establishes the effective range of the radar, and the data recorded during one interval is called a “scan”. Each radar scan will be stored as a column vector of length M, and if N scans are recorded, this will result in a data matrix X of dimension M × N called “Radargram” as presented in Figure 1.

**Figure 2:**
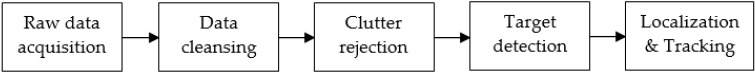
Signal processing steps for target detection and tracking.

The received signal beam includes reflections from both moving and static objects, the noise and direct waves between the transmitting (Tx) and receiving (Rx) antennas. Therefore, the moving-target detection and tracking task is achieved through a number of signal processing phases such as received raw signal preprocessing, background subtraction, target detection, localization and tracking where the output of each step will be the input to the next step as shown in Figure 2. In order to clean our signal and get rid of the noise and cross-talk waves between Tx–Rx, these can be removed or diminished by one suitable bandpass filter as it is explained in [3].

After bandpass-filtering the signal to get rid of the noise, the success in separating the two remaining components of the received signal beam will considerably enhance the accuracy in detection. Different techniques have been developed in this regard, but each one has its drawbacks [4,5], which will be discussed in the next section. The proposed RPCA algorithm efficiently and robustly recovers the low-rank and sparse parts of a large matrix made up of the sum of these two components where the clutter signal is modeled by the low-rank matrix, while the moving target signal is modeled by the sparse matrix component. However, as stated in the previous section, many RPCA models are implemented using batched data [6,7,8], so they require memorization of all samples in order to access every sample in each iteration of the optimization. Storing data first and processing them in a batch manner is not always of great significance, since in most cases we need to record and process frames at the same time. 

On the other hand, there are a few seminal works on online robust PCA [9,10,11,12]. In [9], an incremental and robust subspace learning method is proposed. The method proposes to integrate the M-estimation into the standard incremental PCA calculation. The author defined an incremental way of solving the RPCA problem, where the PCA model updating is performed directly from the previous eigenvectors and a new observation vector. Thus, each newly coming data point is re-weighted by a pre-defined influence function of its residual to the current estimated subspace. However, the performance is only guaranteed if the updated PCA model at each step of an incremental algorithm is good enough to function as the prototype model. In [10], He et al. propose an incremental gradient descent method on Grassmannian manifold for solving the robust PCA problem, named GRASTA. In each iteration, GRASTA uses the gradient of the updated augmented Lagrangian function after revealing a new sample to perform the gradient descent. Balzano et al. [13,14] show that subspace tracking is accomplishable on Grassmannian subspace only when limited entries of data are exposed. In [11], Feng et al. proposed the Online Robust PCA (OR-PCA) which is based on stochastic optimization of an equivalent reformulation of the batch RPCA. OR-PCA processes only one sample per time instance and thus is able to efficiently handle big data and dynamic sample sets, as well as in online systems. Compared to its batch-counterpart where the adopted nuclear norm tightly couples the samples and thus the samples have to be processed simultaneously, OR-PCA pursues the low-rank component in a different manner: using an equivalent form of the nuclear norm, OR-PCA explicitly decomposes the sample matrix into the multiplication of the subspace basis and coefficients plus a sparse noise component. Through such decomposition, the samples are decoupled in the optimization and can be processed separately. However, Feng et al. acknowledged that the computational time of OR-PCA is linear in the sample size and nearly linear in the ambient dimension. The most relatively related to our approach in techniques is [12], where a compressive sensing based recursive robust PCA algorithm is proposed. Their proposed method essentially solves compressive sensing optimization over a small batch of data to update the principal components estimation. However, their algorithm guaranteed a good performance only on data with structured noise which is too restrictive in real life scenarios.

In this work, we demonstrate how the Robust PCA can optimally separate the two subspaces modeling the clutter and the target signals. To tackle the above mentioned RPCA limitations, this work develops a simple processing mechanism based on a relatively small batch of frames and processes the new frames in a fashion that allows to keep the target’s position in real-time. The target detection and localization are highly influenced by the capacity to recover the two subspaces successfully.

This paper considers 2 radar modules, each with a mono-static UWB radar configuration where waveform pulses are transmitted from a single transmitting antenna and the scattered waveforms are received by a collocated receiving antenna. According to our experiments, within a radar range of 10 m, the averaged frame rate is 40 frames per second. Therefore, in order to perform real-time target-detection using the algorithms processing one frame at a time, each frame should be processed in 0.025 s, which is very ideal considering the time required for performing all the involved steps, namely noise filtering, clutter rejection, detecting and localizing the target. For instance, for the stochastic OR-PCA algorithm, the average computational time for one sample of length 240 is 0.0748 s, and 0.0724 s for GRASTA [15].

## 2. UWB Signal Processing Steps

### 2.1. Data Cleansing and Clutter Rejection

The received signal stream X comprises the reflections from physical obstacles within the radar range, and these reflected waves are corrupted with a noise resulting from the target’s motions and the propagation environment. It can be approximated as:

(1) X[n]=E[n]+A[n]+N[n],

where *E*[*n*] is the target signal modeled by the sparse matrix and *A*[*n*] is the clutter signal modeled by the Low rank matrix and *N*[*n*] represents the noise. 

**Figure 3:**
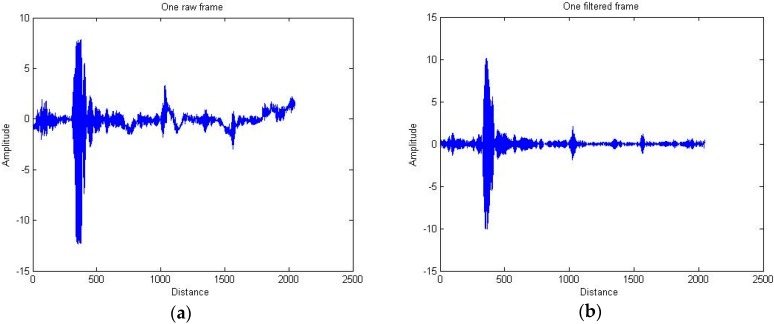
Raw signal frame: (**a**) before bandpass-filtering; (**b**) after bandpass-filtering.

By analyzing the amplitude spectrum, the waves reflected from targets, which are characterized by the dominant amplitude, have been located in the limited middle frequency interval. Other components of the raw signal will be related to noise and direct waves between Tx-Rx. Therefore, the noise and direct waves can be removed by one suitable band-pass filter. Figure 3 shows both the raw and filtered signal frames.

After applying the bandpass filter, the remaining portion of the received signal beam supposedly consists of reflections from the static background and the moving target. The separation of these two components, also known as the background subtraction or clutter rejection, is a key step that aims at removing/minimizing the unwanted clutter signals through which the detection accuracy can be improved. There are a number of approaches for this core step, but they are all sensible to harsh conditions.

#### 2.1.1. Exponential Averaging

In [16], a clutter-reduction method was proposed where the foundational theory of this method is based on exponential averaging. Having an initial estimate of the clutter signal *A*_(*k*−1)_*,* the new estimated clutter signal *A*_(*k*)_ is computed recursively from *A_(k-1)_* and the new incoming radar scan *X_k_* , where k is the time index. Thus, *A*_(*k*)_ is derived as:

(2) $$A_{\left(k\right)\;}=\;\alpha A_{\left(k-1\right)\;}\;+\;\left(1-\alpha \right)\;X_{k\;},$$

where α is a constant scalar weighting factor which is between 0 and 1, *A*_(*k*)_ is the estimated background at *k* time index and *X_k_* is the *k^th^* frame in the dataset. This method is appreciated for its speed for real-time signal processing, but criticized for its very simple clutter estimation leading it to have poor performance in complex scenario cases.

#### 2.1.2. Principal Component Analysis

Computing PCA of a data set *X* involves subtracting the mean of each measurement type and computing the eigenvectors of *XX^T^* [17]. Given the radar-scan data represented by a rectangular matrix *X_ij_*, whose dimension is *M* × *N* (*i =* 1*,* 2*, …, M; j =* 1*,* 2*, …, N*), the N principal components of data matrix X can be given by:

(3) $$Y\;=Z^{T}X,$$

where *X* *= [x*_1_*, x*_2_*, x*_3_*, …, x*_n_*]^T^* is the zero-mean input vector, *Y* *= [y*_1_*, y*_2_*, y*_3_, *…, y_n_]^T^* is the output vector called the vector of principal components, *Z* is an *M × N* matrix that transforms *X* into *Y*.·Z can be computed using the covariance matrix. PCA assumes that *Z* is an orthonormal matrix ($Z_{i}^{T}$*·Z _j_* = *δij*) such that the covariance matrix of *Y*; (*C_y_*) is diagonalized. Then, the covariance matrix *C_x_* of *X* is given by:

(4) $$C_{x}=\frac{1}{N}XX^{T}.$$

The eigenvector and eigenvalue matrices of *C_x_*, denoted *Φ* and *Λ* respectively, are computed by:

(5) $$C_{x}\Phi \;=\;\Phi \Lambda ,$$

where Λ = diag(*λ*_1_*, λ*_2_*, λ*_3_, *…, λ_N_*) and *λ*_1_*, λ*_2_*, λ*_3_, *…, λ_N_* are the eigenvalues. If one assumes that the eigenvalues are sorted in an ascending order, *λ*_1_ *≥ λ*_2_ *≥ λ*_3_ *≥…≥ λ_N_*, then the N-leading eigenvector-matrix *Z* is given by:

(6) $$Z\;=\left[\Phi_{1}\;,\;\Phi_{2}\;,\;\Phi_{3}\;,\;\ldots ,\;\Phi_{N}\;\right]\;.$$

The clutter-free signal space can be extracted from the original entire signal space according to

(7) $$E\;=Z_{n}X\;.$$

We select the matrix *Z_n_* to be a matrix whose rows *z*_n_ are the eigenvectors of *XX^T^* (the principal components of *X*).

#### 2.1.3. RPCA via Inexact ALM

The above-defined classical PCA aims at the exact recovery problem from corrupted low-rank data owing to small errors and noise, but it cannot effectively deal with incomplete or missing real-world data suffering greater corruption. Therefore, the robust principle component analysis (RPCA) has been proposed for corrupted low-rank data recovery [18,19,20,21,22,23].

Recently, Wright et al. [24] have shown that under rather broad conditions the answer is affirmative: as long as the error matrix *E* is sufficiently sparse (relative to the rank of *A*), one can exactly recover the low-rank matrix *A* from *X = A* + *E* by solving the following convex optimization problem:

(8) $$\underset{A,\;E}{min}{\left|\left|A\right|\right|}_{*\;}+\;\lambda {\left|\left|E\right|\right|}_{1\;},\text{ subject to }X=A+E,$$

where ∥⋅∥_∗_ is the nuclear norm of a matrix, and thus the sum of its singular values, ∥⋅∥_1_ is the sum of the absolute values of matrix entries, and λ is a positive weighting parameter on the sparse error.

Different algorithms have been recently developed to solve the RPCA problem. They all model the background with a low rank subspace, while the moving foreground objects constitute the correlated sparse outliers.

As stated in the first section of this paper, the proposed model follows the techniques developed by Augmented Lagrange Multiplier. Given a radar-scan data matrix *X* whose dimensions are M × N, and a weight λ on sparse error term, if we identify:

(9) $$\{\begin{array}{c} X=(A,E),\\ f(X)={∥A∥}_{*}+\lambda {∥E∥}_{1}\\ h(X)=X-A-E \end{array},$$

where *A* and *E* are the low-rank and sparse matrices respectively. The general augmented Lagrangian function will then be defined as follows:

(10) $$L\left(X,\;Y,\;\mu \right)=\;f\left(X\right)+\langle \;Y,\;h\left(X\right)\rangle +\;\frac{\mu }{2}{\left|\left|h\left(X\right)\right|\right|}_{F}^{2}\;,$$

where *μ* is a positive scalar. It has been proven in [25] that if *μ* is increasingly changing in each iteration and f and h are continuously differentiable functions, then under rather general conditions the ALM method converges Q-linearly toward the optimal solution. 

In [11] Feng et al. explained how the use of RPCA algorithm for online processing is challenged by the nuclear norm defined in Equation 8. In order to find the minimum nuclear norm, a Singular Value Decomposition (SVD) has to be performed across all samples, though SVD is the operation that consumes the most running time. In [26], the inexact ALM (IALM) approach was defined and converged to global minimum by computing only the first few, largest singular values. 

The inexact ALM method will then be defined by Algorithm 1:
**Algorithm 1** Robust PCA via the Inexact ALM Method   1: *Y_0_ = X/J(X); E_0_ = 0; μ_0_ > 0; ρ> 1; k = 0*   2: while not converged do     3: *(U,S,V) = svd(X − E_k_ +*$\mu_{k}^{-1}Y_{k}$*)*     4: *A_k+1_ =U*$S_{\mu_{k}^{-1}}\;\left[S\right]V^{T}$     5: *E_k+1_=*
$S_{\lambda \mu_{k}^{-1}}\;\left[X-\;A_{k+1}+\;\mu_{k}^{-1}Y_{k}\right]$     6: *Y_k+1_ = Y_k_ +μ_k_ (*$X\;-A_{k+1\;}$
−
*E_k+1_)_;_*
      *μ_k+1_ = ρμ_k_*
     7: *k = k + 1*   8: end while

The algorithm takes *X* (an *MxN* radar-Scans matrix) and λ (a positive weighting parameter on the sparse error) as inputs and returns *A_k_, E_k_*: the estimated background and foreground respectively.

In [14] the value of λ is said to be calculated as $\frac{1}{\sqrt{max\left(M,N\right)}}\;$, but our extensive experiments proved that if *M>>N* then $\lambda =\frac{1}{\sqrt{M/N}}$ (or vice-versa) will be used; otherwise, the first holds.

From Theorem 1 and Theorem 2 elaborated in [26], we can get the following conclusion: for geometrically growing *μ_k_*, IALM converges Q-linearly, and the faster *μ_k_* grows, the faster IALM converges. However, when *ρ* is too large such that the condition $\underset{k\to +\infty }{lim}\mu_{k}^{\;}{\left(E_{k+1}-E_{k}\right)}^{\;}=0$ is violated, IALM may fail to converge to the optimal solution. Thus, if seeking both optimality and fast convergence, the values of *μ_0_* and *ρ* must be chosen cautiously. 

### 2.2. Target Detection

The target detection task implies analyzing a signal frame and deciding if there is a target within the radar range or not which can be solved using methods of statistical decision theory. Those methods will take a clutter-free signal and compare it with a given threshold to reach such a decision. Besides the simple threshold detectors, the interperiod-correlation processing (IPCP) detector [27], the constant false alarm rate (CFAR) [28], the CLEAN detection algorithm [29] and their modifications have shown their capacity to provide good and robust results in target detection by UWB radar. 

In this regard, with the intention to illuminate the differences in performance of the discussed background techniques, their respective outputs were passed to the CLEAN algorithm for target detection and the obtained results are compared.

However, the geometric attenuation of the wave emitted by the antenna must be considered and its relation with the distance between the radar and a reflecting point [30]. 

#### 2.2.1. Path Loss Compensation 

The radar range equation (or simply radar equation) associates the ratio between the received power and the transmitted power with the forth power of the inverse distance to a reflecting point:

(11) $$\frac{P_{rec}}{P_{T}}=\frac{G_{T}\;A_{e}\;\sigma }{{\left(4\pi \right)}^{2}R^{4}\;L}\;,$$

where *P_rec_* is the power captured by the receiving antenna, *P_T_* is the transmitted power, *G_T_* is the gain of transmitting antenna, *A_e_* is the effective aperture of the receiving antenna, *σ* is the radar section, *R* is the range (target distance), and *L* denotes all losses affecting the radar performance.

In the development of this range equation, it is assumed that both the radar’s transmitter and the target are isotropic radiators. An isotropic radiator does not focus energy; instead it distributes power uniformly over the surface of a sphere centered on the antenna. We note that an isotropic radiator cannot exist in the “real world”. However, it is a mathematical and conceptual concept that is often used in radar theory.

Another note is that the development of the above equation makes the tacit assumption that the antenna is pointed exactly at the target, which is not always the case depending on the various positions of the target; otherwise, GT must be modified to account for this.

All these mathematical assumptions used in the radar theory accept the flexibility in the range power, which can also vary according to the target’s physical shape and properties [31]. In this regard, following our experimentation, we have chosen 2 as a power of the range so that the path loss will be compensated with the squared distance of a reflecting point. Therefore, the signal’s power at each point was weighted with *R^2^* where *R* is the range between the radar and that particular point.

#### 2.2.2. CLEAN Algorithm

After the clutter-free signal is amplified for the path loss compensation, the resulting signal will be the input of the CLEAN algorithm for target detection. The CLEAN algorithm is defined by Algorithm 2:
**Algorithm 2** CLEAN Algorithm(1) **Input:**
*v(k):* Waveform template; *T_clean_ :* detection threshold*, x(t)*: data frame (2) **Initialize:** Form initial residual waveform *d_0_(t)* = *x(t)*. Set iteration counter *i = 0*.(3) **Signal analysis:** Compute cross-correlation *r_vd_(τ)* between *v(t)* and *d_i_(t);* The time-index associated to the maximum magnitude of *r_vd_(τ)* is the *i^th^* estimated TOA:  *ñi(t) = argmaxτ |rvd(τ)|*. The cross-correlation at ñi(t) is the ith estimated amplitude: ãi(t) = rvd(ñi(t)). If the *ã_i_(t)> T_clean_*:  a **Increment the iteration counter:**
*i*
*←*
*i +* 1*.*  b **Update residual waveform:**
*d_i_(t) = d_i−1_(t) − ã_i_(t)v(t − ñ_i_(t)).*  c **Iterate:** Go to step 3.(4) Stop

Thus, the CLEAN Algorithm will give one TOA for every target, even when it is a distributed target. This TOA will be used to calculate the position (distance) of the target.

R(t) = ñ(t) × c/2, (12)

where *R* denotes the distance between the radar and the target, *ñ* is the *TOA, c* is the speed of light and *t* denotes the observation time which is herein associated with the frame number. 

### 2.3. Target Localization

**Figure 4:**
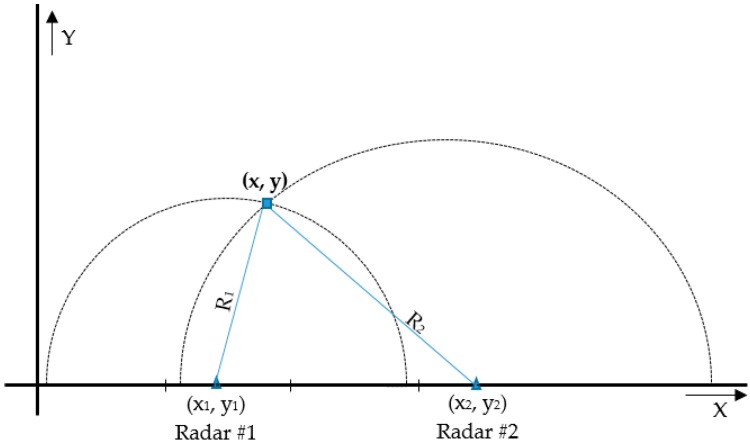
2D positioning with two radars.

The intention of this step is to find the coordinates of the target’s position in a 2D space. Using a mono-static UWB radar, we can only estimate a 1D-positioning which gives the distance *R* between the radar and the target. However, there are many points laying at the same distance from the radar but in different positions, and these make up the circumference of a circle whose radius is *R*. In order to obtain a 2D-positioning, we need at least two receiving antennas [32] or two mono-static radars. The distance between the two modules and their respective positions is crucial to the accuracy of the target-positioning [33]. For this regard in this work we estimate the 2D coordinates of the target using two independent monostatic radars (connected on a same PC for real-time processing). These radars are capturing one frame each at a time consecutively to avoid cross-talk between them and to allow them to capture frames in relatively close times. Data from the respective radar modules are stored as two different Radargrams which will be processed independently and will result in series of (*R_i_, t*) couples where *R* is the distance between the radar and the target, *i* = 1, 2 denotes the radar’s index and *t* is the observation time which is herein associated with the frame number. Assuming that the coordinates of the radars are known and are (*x_i_, y_i_*) (*i* = 1, 2), the 2D coordinates of the target position will be given by a geometric interpolation presented in Figure 4. 

### 2.4. Overlapping Windows Processing

Our particular motivation for this work is to exploit the robustness of RPCA methods that have always been criticized for being batch-oriented and inefficient for online processing systems or processing incremental data sets. We call these methods for clutter removal in the task of detecting and localizing a moving target in real-time using an IR-UWB radar.

**Figure 5:**
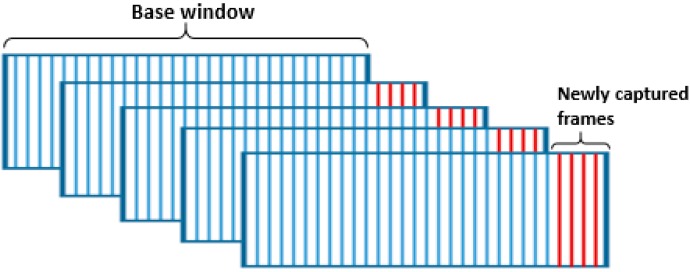
Overlapping windows processing framework.

**Figure 6:**
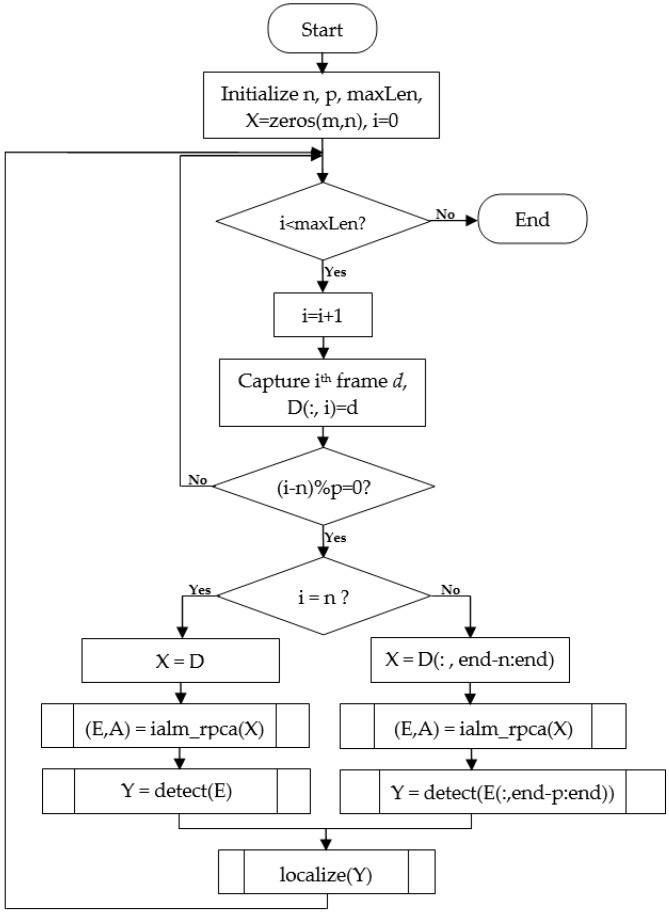
Overlapping windows method flowchart.

The proposed method essentially follows the normal IALM to process a small batch of data which is continuously updated as new frames are captured. This framework is shown in Figure 5. The method considers a small batch of *n* frames as one “window.” The first window, called the “base window,” will be recorded first. This window will go through the processing steps, including data cleansing, background subtraction, target detection and localization. The next window is generated by appending the *p* newly recorded frames to the previous window and eliminating the *p* first frames of the window to keep the same window size. The new window will be processed in the same way as the base window, but for the detection task only the p new frames will be concerned. The flowchart in Figure 6 shows the working mechanism of this method. 

The choice of *n* (window’s width) and *p* (shift step) are crucial and must be done counting on their effects on the overall performance of the system. In instance, a very large value of *n* will result in a very big dataset (window) and hence slow down the processing, and also we will have to wait longer to get the base window. In contrast, a very small *n* will not allow RPCA to converge to its optimal solution. On the other hand, a great value of *p* will devastate our “in real-time” concept. 

The radar module of this work has an average frame rate of 40 frames per second. Therefore, we chose *n =* 20 and *p* = 4. The value of n was decided basing on experimental observations, thus the minimum number of frames that allows us to reach the optimal solution. Each window is processed in approximately 0.1 s, so we chose *p =* 4 for time compensation since 0.1 s is the time required to capture 4 frames.

## 3. Experimental Results

The experiments of this work were conducted in indoor conditions, in a building’s pavilion of approximate size 8 m × 10 m. The two radars were set up on a table of 1 m high, 2.5 m distant from each other and connected with a computer that was running the program. Numerous experiments were run with different scenarios, namely with 1, 2, 3 and 5 persons, to prove the consistent efficiency of the proposed method. In all these scenarios, the targets were moving with an average velocity of 10 m/12 s. 

**Figure 7:**
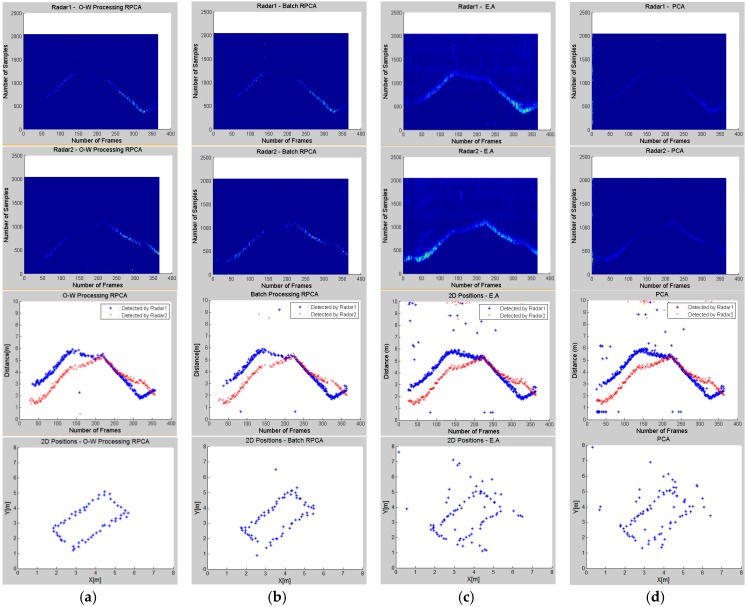
Clutter-free signals of the 2 radars, their respective detection results and the resulting 2D positioning given by different clutter removal techniques: Column (**a**) O-W processing RPCA; Column (**b**) Batch Processing RPCA; Column (**c**) EAM; Column (**d**) PCA.

While running the experiment with online processing, the raw signal frames were saved in order to be able to process the same scenario later with other methods for a better comparison. Figure 7 shows the results of the respective clutter rejection methods. 

As discussed in the sections above, the raw frames recorded by each radar are processed separately for the background removal. The first and second rows of Figure 7 present the clutter-free signals of the two radar modules using different clutter removal algorithms. It is clear that the reflected waves from farther targets are very weak, henceforth the need of path loss compensation. 

The estimated clutter-free signals of both radar modules are submitted to the target-detection algorithm and the results of both datasets are plotted in the third line of Figure 7. At this stage, the results are already elaborated for comparison. If we compare these methods, we can see the weaknesses of PCA and Exponential Averaging in separating the two subspaces, which results in many false alarms in the detection step. The detection results from the two radars will then be used for the target-localization task, and the results are shown in the last row of Figure 7.

The results in Figure 7 present the outcome of the target detection and localization steps based on the clutter removal results of every method. We can clearly see the good performance of the first two methods, RPCA in both live and batched data processing, and this can also be proved by the numeric results presented in Table 1. For a better comparison, we use the following metrics to explain the differences in performance of these methods:

✓ RMSE (Root Mean Square Error): the commonly used metric to measure the differences between two data sets, one predicted by a model or an estimator and the actually observed one. In here, it is the average of the individual RMSEs between each radar’s estimated clutter and the true clutter. The true clutter is approximated by recording a relatively large number of frames in a target-free space, and these frames are averaged to minimize the effect of the noise.
  (12) $$RMSE=\frac{1}{M}\sum_{i=1}^{M}(\sqrt{\frac{1}{N}\sum_{J=1}^{N}{\left(A_{\left(i,j\right)}-{\overset{⌢}{A}}_{\left(i,j\right)}\right)}^{2}}),$$
  where A is the estimated clutter and Ȃ is the true clutter.
✓ Localization error: the average distance-error (the Euclidean distances) between the estimated target position and the approximated the true path of the target. We assume the target was moving following straight lines.

**Figure 8:**
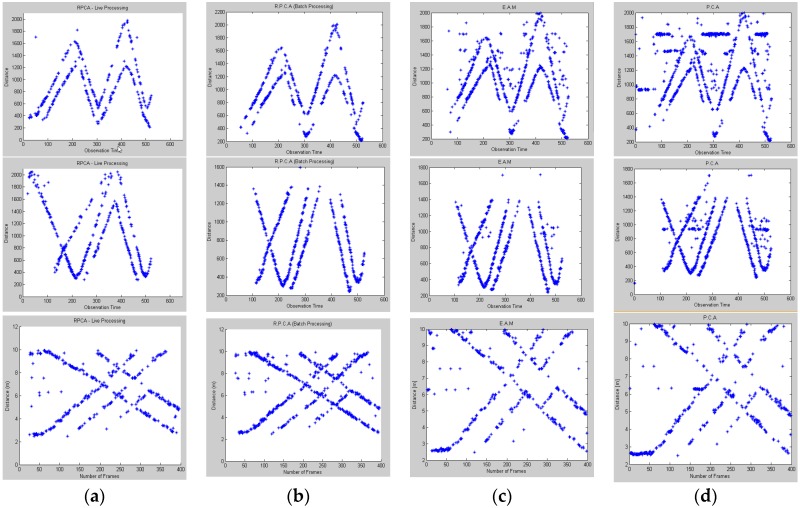
Target detection results for 2, 3 and 5 targets-scenarios: column (**a**) O-W processing RPCA, column (**b**) batch processing RPCA, column (**c**) EAM, column (**d**) PCA.

Other experiments, of which the results are shown in Figure 8, were run with 2, 3 and 5 moving targets, and the processing procedure took the same flow as in a one-person scenario. The presented results show the detection results from one radar’s data, and are numerically compared based only on RMSE as it is presented in Table 2.

## 4. Conclusions

The task of detecting and localizing a moving target using IR-UWB radar requires a perfect separation of the waves scattered by the time-invariant background from the waves scattered by the moving targets. Subspace learning has proved its capacity to recover the low-rank matrix modeling the clutter signal and the sparse error matrix modeling the target signal.

In this paper, Robust PCA via the Inexact Augmented Lagrange Multiplier is proposed for clutter removal in IR-UWB-based target detection and localization, and a novel processing mechanism is developed to adapt this batch-oriented algorithm to the real-time processing trend. The algorithm performs well in simple scenarios as well as in complex ones. The results prove that we can accurately perform the task of detection and localizing a moving target within an online framework. 

## Figures and Tables

**Table 1:** Numeric results for a one-person-scenario.

	RPCA (Live)	RPCA (Batch)	EAM	PCA
RMSE	1.261	1.287	1.873	1.962
Locat. Error (m)	0.26	0.49	1.33	1.29

**Table 2:** Numeric results (RMSE) for a multiple-person scenario.

	RPCA (Live)	RPCA (Batch)	EAM	PCA
2 Moving targets	3.715	4.828	5.489	6.712
3 Moving targets	3.712	4.734	5.00	6.104
5 Moving targets	1.397	1.346	1.576	1.958

## Abbreviations

IR	Impulse Radio
UWB	Ultra-Wide Band
PCA	Principal Component Analysis
RPCA	Robust Principal Component analysis
IALM	Inexact Augmented Lagrange Multiplier
EA	Exponential Averaging
O-W	Overlapping Windows
EMW	Electromagnetic wave
Tx	Transmitting Antenna
Rx	Receiving Antenna
SVD	Singular Value Decomposition
TOA	Time of arrival
